# Increased Risk of Ischemic Stroke after Hyperosmolar Hyperglycemic State: A Population-Based Follow-Up Study

**DOI:** 10.1371/journal.pone.0094155

**Published:** 2014-04-08

**Authors:** Jen-Yu Wang, Cheng-Yi Wang, Yung-Sung Huang, Pin-Fan Chen, Kuang-Yung Huang, Pesus Chou, Wei-Cheng Lian, Ching-Chih Lee

**Affiliations:** 1 Department of Internal Medicine, Cardinal Tien Hospital, School of Medicine, Fu-Jen Catholic University, New Taipei City, Taiwan; 2 Division of Neurology, Department of Internal Medicine, Buddhist Dalin Tzu Chi General Hospital, Chiayi, Taiwan; 3 Division of Metabolism and Endocrinology, Department of Internal Medicine, Buddhist Dalin Tzu Chi General Hospital, Chiayi, Taiwan; 4 School of Medicine, Tzu Chi University, Hualian, Taiwan; 5 Division of Allergy, Immunology, and Rheumatology, Department of Internal Medicine, Buddhist Dalin Tzu Chi General Hospital, Chiayi, Taiwan; 6 Community Medicine Research Center and Institute of Public Health, National Yang-Ming University, Taipei, Taiwan; 7 Department of Otolaryngology, Buddhist Dalin Tzu Chi General Hospital, Chiayi, Taiwan; 8 Department of Education, Buddhist Dalin Tzu Chi General Hospital, Chiayi, Taiwan; 9 Center for Clinical Epidemiology and Biostatistics, Buddhist Dalin Tzu Chi General Hospital, Chiayi, Taiwan; Massachusetts General Hospital, United States of America

## Abstract

**Background:**

Although much attention has been focused on the association between chronic hyperglycemia and cerebrovascular diseases in type 2 diabetes mellitus (DM) patients, there is no data regarding the risk of ischemic stroke after a hyperosmolar hyperglycemic state (HHS) attack. The objective of this study was to investigate the risk of ischemic stroke in type 2 DM patients after an HHS attack.

**Methods:**

From 2004 to 2008, this retrospective observational study was conducted on a large cohort of Taiwanese using Taiwan’s National Health Insurance Research Database (NHIRD). We identified 19,031 type 2 DM patients who were discharged with a diagnosis of HHS and 521,229 type 2 DM patients without an HHS diagnosis. Using the propensity score generated from logistic regression models, conditional on baseline covariates, we matched 19,031 type 2 DM patients with an HHS diagnosis with the same number from the comparison cohort. The one-year cumulative rate for ischemic stroke was estimated using the Kaplan-Meier method. After adjusting covariates, Cox proportional hazard regression was used to compute the adjusted one-year rate of ischemic stroke.

**Results:**

Of the patients sampled, 1,810 (9.5%) of the type 2 DM patients with HHS and 996 (5.2%) of the comparison cohort developed ischemic stroke during the one-year follow-up period. After adjusting for covariates, the adjusted HR for developing ischemic stroke during the one-year follow-up period was 1.8 (95% C.I., 1.67 to 1.95, P<0.001) for type 2 DM patients with HHS compared with those without HHS.

**Conclusion:**

Although DM is a well-recognized risk factor for atherosclerosis, type 2 DM patients that have suffered a HHS attacks are at an increased risk of developing ischemic stroke compared with those without HHS.

## Introduction

Atherosclerosis, with its complications of ischemic stroke and coronary artery diseases, is responsible for the highest morbidity and mortality in patients with type 2 diabetes mellitus (DM) [1]. The metabolic abnormalities that accompany type 2 DM, (particularly chronic hyperglycemia, dyslipidemia, and insulin resistance) impair normal functioning of endothelial cells, vascular smooth muscle cells, and platelets which render arteries in type 2 DM patients susceptible to atherosclerosis [2] and increase the likelihood of ischemic stroke.

In particular, acute hyperglycemia has been shown to worsen the size of the infarct in ischemic stroke [3], [4] and retrospective studies in humans have shown that hyperglycemia on admission may worsen the clinical outcome in ischemic stroke [5], [6]. Hyperglycemic crisis may also be associated with an increased risk of stroke. The hyperosmolar hyperglycemic state (HHS) is typically characterized by hyperglycemia and dehydration caused by osmotic diuresis, which leads to hyperviscosity and a hypercoagulable state [7], [8]. Besides ischemic stroke, complications include myocardial infarction, pulmonary embolism and mesenteric vessel thrombosis [9], [10].

Although most studies have demonstrated that hyperglycemia on admission is associated with increased risk of ischemic stroke [11], there is no data regarding the post-discharge risk of ischemic stroke after an HHS attack in type 2 DM patients. The objective of this study was to investigate the risk of ischemic stroke after an HHS attack in type 2 DM patients using Taiwan’s National Health Insurance Research Database (NHIRD).

## Materials and Methods

### Ethics Statement

This study was approved by the Institutional Review Board of Buddhist Dalin Tzu Chi General Hospital, Taiwan. Since all identifying personal information was removed from the secondary files prior to analysis, the review board waived the requirement for written informed consent from the patients involved.

### Database

From 2004 to 2008, this retrospective observational study was conducted on a large cohort of Taiwanese using Taiwan’s NHIRD. The National Health Insurance program, which provides compulsory universal health insurance, was implemented in Taiwan in 1995. It enrolls up to 99% of the Taiwanese population and contracts with 97% of all medical providers. The database contains comprehensive information on insured subjects, including dates of clinical visits, diagnostic codes, details of prescriptions, and expenditure amounts.

Our study cohort consisted of all type 2 DM patients in Taiwan with International Classification of Diseases, Ninth Revision, Clinical Modification (ICD-9-CM) codes, 250.X0 or 250.X2, who had been hospitalized for HHS (ICD-9-CM, 250.20 or 250.22) between 2004 and 2008, compared to hospitalized type 2 DM patients without HHS. We excluded those patients with any type of stroke (ICD-9-CM, 430–438) diagnosed before or during the index hospitalization.

Each patient was tracked for a one-year after his or her first discharge to identify outcomes, including ischemic stroke diagnosis codes (ICD-9-CM, 430, 431, 433.x1, 434.x1, or 436.x). The NHIRD has an accuracy of up to 95% in recording diagnoses of ischemic stroke [12]. To maximize case ascertainment, only patients hospitalized for cerebrovascular events were included. These patients were then linked to the administrative data for the period from 2004–2008 to calculate cerebrovascular disease-free survival time, with cases censored for patients who drew back guarantees from the National Health Insurance Program or were still robust without defined events at the end of follow-up.

### Variables

Patient characteristics included age, gender, co-morbidities, geographic location, level of urbanization, and socioeconomic status (SES), as previous studies have shown that the incidence of ischemic stroke was associated with geographic location, level of urbanization, and SES [13]–[15]. Co-morbid conditions, such as coronary artery disease, atrial fibrillation, dyslipidemia, and chronic renal failure, were identified according to the ICD-9-CM system.

Patients were classified into two sub-groups: (1) high individual SES (civil servants, regular or full-time paid personnel with a government affiliation, employees of privately owned institutions), and (2) low individual SES (self-employed individuals, members of the farmers’ or fishermen’s associations, other employees, military veterans, substitute service draftees, and members of low-income families) [16]. The urbanization level of residences were classified in seven levels based on five indices in Taiwan: (1) population density, (2) percentage of residents with college level or higher education, (3) percentage of residents above 65 years old, (4) percentage of residents who were agriculture workers, and (5) the number of physicians per 100,000 people [17]. We recorded the urbanization level of residences as urban (urbanization level 1), sub-urban (urbanization levels 2–3), or rural (urbanization levels 4–7). The geographic regions where the DM patients resided were subdivided into northern, central, southern and eastern Taiwan.

### Statistical Analyses

The SPSS (version 15, SPSS Inc., Chicago, IL, USA) was used for data analysis. Propensity score stratification was applied to replace the wide host of confounding factors that may be present in an observational study with such variable factors. Pearson’s chi-square test was used for categorical variables such as gender, comorbidities, level of urbanization, geographic regions of residence, and SES. Continuous variables were analyzed with a one way ANOVA test. The Cox proportional hazards regression model, adjusting for patients’ characteristics (age, gender, Charlson Comorbidity Index Score, urbanization, area of residence and SES) was used to compare outcomes between the two cohorts. The one-year cumulative rate of ischemic stroke was estimated using the log rank test to examine the differences in the risk of developing ischemic stroke between type 2 DM patients with and without HHS. We calculated hazard ratios (HR) along with 95% confidence intervals (C.I.) using a significance level of 0.05. A two-sided p-value (*P*<0.05) was used to determine statistical significance.

## Results

We identified 19,031 type 2 DM patients who were discharged with a diagnosis of HHS and 521,229 type 2 DM patients without a HHS diagnosis. Using the propensity score generated from logistic regression models, conditional on baseline covariates, we matched 19,031 type 2 DM patients with an HHS diagnosis with the same number from the comparison cohort. The demographic characteristics and selected morbidities for the two cohorts are shown in Table 1.

**Table 1:** Characteristics in type 2 DM patients,2004–2008.

Characteristics	Before matched			Propensity score-matched		
	Patients withoutHHS	Patients withHHS	*p* value	Patients withoutHHS	Patients withHHS	*p* value
Patient no.	521229	19031		19031	19031	
Mean age (s.d.), year	64.5±13.6	66.0±14.7	<0.001	66.1±13.3	66.1±14.7	0.80
Male gender	269273(51.7)	10444(54.9)	<0.001	10476(55.0)	10444(54.9)	0.74
*Comorbidity*						
Hypertension	293150(56.2)	10976(57.7)	<0.001	10889(57.2)	10976(57.7)	0.37
Coronary artery disease	120287(23.1)	5732(30.1)	<0.001	5750(30.2)	5732(30.1)	0.84
Atrial fibrillation	22137(4.2)	1090(5.7)	<0.001	1089(5.7)	1090(5.7)	0.98
Hyperlipidemia	67296(12.9)	3300(17.3)	<0.001	3255(17.1)	3300(17.3)	0.54
Chronic renal failure	37583(7.2)	2565(13.5)	<0.001	2397(12.6)	2565(13.5)	0.01
*Socioeconomic status*			<0.001			
High	159115(30.5)	4949(26.0)		4934(25.9)	4949(26.0)	0.88
Moderate	234088(44.9)	9005(47.3)		8976(47.2)	9005(47.3)	
Low	128026(24.6)	5077(26.7)		5121(26.9)	5077(26.7)	
*Geographic region*			<0.001			0.98
Northern	260703(50.0)	8547(44.9)		8575(45.1)	8547(44.9)	
Central	93495(17.9)	3784(19.9)		3771(19.8)	3784(19.9)	
Southern	149458(28.7)	6146(32.3)		6121(32.2)	6146(32.3)	
Eastern	17573(3.4)	554(2.9)		564(3.0)	554(2.9)	
*Urbanization level*			<0.001			0.40
Urban	167143(32.1)	7164(37.7)		7159(37.6)	7164(37.7)	
Suburban	223195(42.8)	7811(41.0)		7913(41.6)	7811(41.0)	
Rural	130891(25.1)	4056(21.3)		3959(20.8)	4056(21.3)	

**Figure 1:**
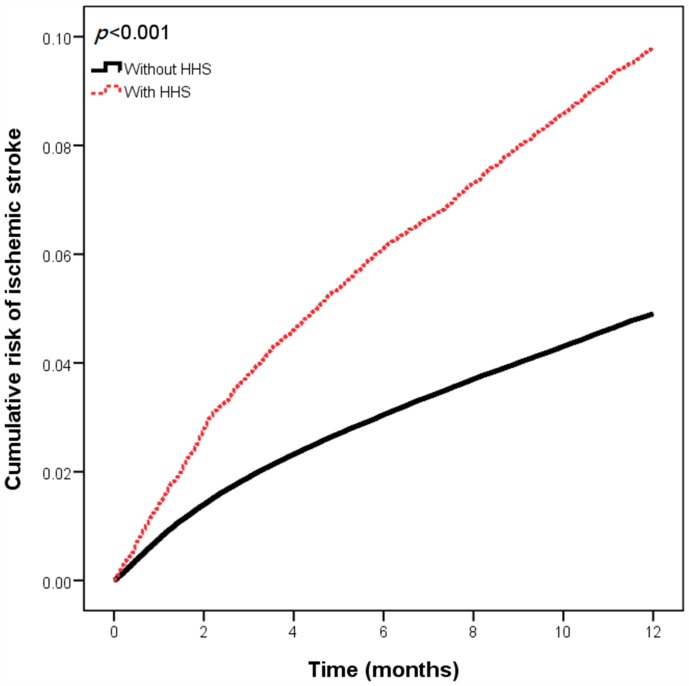
Cumulative risk of ischemic stroke for the DM patients with HHS and those without HHS.

During the one-year follow-up period, 1,810 (9.5%) type 2 DM patients with HHS and 996 (5.2%) patients from the comparison cohort developed ischemic stroke (Table 2). The HRs and 95% C.I.s of the various risk factors for ischemic stroke are presented in Table 3. Age, hypertension, coronary artery disease, atrial fibrillation, hyperlipidemia, chronic kidney disease, and moderate SES were associated with an increased risk of ischemic stroke. After adjusting for the covariates, the adjusted HR for developing ischemic stroke during the one-year follow-up period was 1.8 (95% C.I., 1.67 to 1.95, *P*<0.001) for type 2 DM patients with HHS compared with those without HHS. Figure 1 shows the Kaplan-Meier failure curve of developing ischemic stroke in type 2 DM patients with and without HHS. The type 2 DM patients with HHS had a significantly higher one-year cumulative rate of ischemic stroke than patients in the comparison cohort (*P*<0.001), after adjusting for the covariates.

**Table 2:** The cumulative rate of ischemic stroke in type 2 DM patients with and without HHS.

Characteristics	6 months			1-year		
	*n*	Events (%)	*p* value	*n*	Events (%)	*p* value
*Ischemic stroke*						
Before matched			<0.001			<0.001
Without HHS	521229	15391 (3.0)		521229	24042(4.6)	
With HHS	19031	1143 (6.0)		19031	1810(9.5)	
Propensity score-matched			<0.001			<0.001
Without HHS	19031	631(3.3)		19031	996(5.2)	
With HHS	19031	1143(6.0)		19031	1810(9.5)	

**Table 3:** Hazard ratios for the ischemic stroke in DM patients with and without HHS.

Characteristics	Before matched		Propensity score-matched	
	Hazard ratio (95%CI)	*p* value	Hazard ratio (95%CI)	*p* value
*Univariate model*				
Without HHS	1		1	
With HHS	2.05(1.96–2.15)	<0.001	1.81(1.68–1.96)	<0.001
*Multivariable regression model* *				
Without HHS	1		1	
With HHS	1.75(1.67–1.84)	<0.001	1.80(1.67–1.95)	<0.001
Age	1.04(1.03–1.04)	<0.001	1.03(1.02–1.03)	<0.001
*Gender*				
Male	1		1	
Female	0.90(0.88–0.92)	<0.001	0.96(0.89–1.04)	0.32
*Comorbidity*				
Hypertension	2.55(2.47–2.64)	<0.001	2.40(2.18–2.64)	<0.001
Coronary artery disease	1.49(1.45–1.52)	<0.001	1.42(1.31–1.53)	<0.001
Atrial fibrillation	1.55(1.48–1.61)	<0.001	1.29(1.14–1.46)	<0.001
Hyperlipidemia	1.38(1.34–1.43)	<0.001	1.15(1.05–1.27)	0.004
Chronic renal failure	1.50(1.45–1.56)	<0.001	1.25(1.14–1.38)	<0.001
*Socioeconomic status*				
High	1		1	
Moderate	1.06(1.02–1.09)	0.001	1.12(1.01–1.24)	0.03
Low	1.08(1.04–1.12)	<0.001	1.06(0.95–1.18)	0.29
*Geographic region*				
Northern	1		1	
Central	1.20(1.16–1.25)	<0.001	1.20(1.09–1.33)	<0.001
Southern	1.08(1.05–1.12)	<0.001	1.02(0.93–1.13)	0.69
Eastern	0.95(0.88–1.02)	0.16	0.91(0.72–1.16)	0.45
*Urbanization level*				
Urban	1		1	
Suburban	0.94(0.91–0.98)	<0.001	0.99(0.91–1.09)	0.89
Rural	0.87(0.84–0.91)	<0.001	0.97(0.86–1.09)	0.61

*Adjusted for patient age, gender, Charlson Comorbidity Index Score, geographic region, urbanization level, hospital ownership, and comorbidity.

Among the type 2 DM patients with HHS, predictors of ischemic stroke were analyzed using the Cox regression model. In univariate analysis, age, hypertension, coronary artery disease, atrial fibrillation, chronic kidney disease, moderate SES and residence in central Taiwan were associated with increased risk for ischemic stroke. Increased age (HR = 1.03; 95% C.I., 1.02 to 1.03), hypertension (HR = 2.36; 95% C.I., 2.10 to 2.66), coronary artery disease (HR = 1.39, 95% C.I., 1.26 to 1.52), atrial fibrillation (HR = 1.24; 95% C.I., 1.10 to 1.41), chronic renal failure (HR = 1.22; 95% C.I., 1.08–1.38), moderate SES (HR = 1.20; 95% C.I., 1.05 to 1.36), and residence in central Taiwan (HR = 1.24; 95% C.I., 1.10 to 1.41) remained independent risk factors for ischemic stroke using the multivariable model (Table 4).

**Table 4:** Hazard ratios for the ischemic stroke in HHS patients.

Characteristics	Univariate model			Multivariable regression model*		
	HR	95% CI	*p* value	HR	95% CI	*p* value
Age	1.03	1.02–1.03	<0.001	1.03	1.02–1.03	<0.001
*Gender*						
Male	1			1		
Female	1.29	1.18–1.42	0.03	1.01	0.92–1.11	0.84
*Comorbidity*						
Hypertension	2.94	2.62–3.29	<0.001	2.36	2.10–2.66	<0.001
Coronary artery disease	1.85	1.69–2.03	<0.001	1.39	1.26–1.52	<0.001
Atrial fibrillation	1.82	1.56–2.13	<0.001	1.24	1.06–1.46	0.007
Hyperlipidemia	1.04	0.92–1.17	0.51	1.11	0.98–1.25	0.11
Chronic renal failure	1.46	1.30–1.64	<0.001	1.22	1.08–1.38	0.001
*Socioeconomic status*						
High	1			1		
Moderate	1.31	1.16–1.47	<0.001	1.20	1.05–1.36	0.007
Low	1.20	1.05–1.37	0.007	1.08	0.94–1.23	0.29
*Geographic region*						
Northern	1			1		
Central	1.29	1.14–1.45	<0.001	1.24	1.10–1.41	0.001
Southern	1.00	0.90–1.12	0.94	0.99	0.87–1.12	0.99
Eastern	0.99	0.74–1.32	0.95	0.93	0.70–1.25	0.93
*Urbanization level*						
Urban	1			1		
Suburban	0.90	0.81–1.00	0.05	1.03	0.92–1.15	0.67
Rural	0.88	0.77–0.99	0.04	1.05	0.91–1.22	0.50

*Adjusted for patient age, gender, Charlson Comorbidity Index Score, geographic region, urbanization level, hospital ownership, and accreditation level.

## Discussion

Compared with type 2 DM patients without HHS, we found that type 2 DM patients that had suffered an HHS attack were at an increased risk of developing subsequent ischemic stroke (HR, 1.8; 95% C.I., 1.67 to 1.95, P<0.001). Using a multivariable model, we also found that increased age, hypertension, coronary artery disease, atrial fibrillation, chronic renal failure, moderate SES, and residence in central Taiwan were independent risk factors for ischemic stroke.

DM has been shown to be an important risk factor for ischemic stroke. In the Multiple Risk Factor Intervention Trial (MRFIT) [18], the risk of stroke was three times higher in DM subjects. However, data regarding the risk of ischemic stroke after stabilization following an HHS attack are lacking. According to the one-year follow-up data, the risk of developing ischemic stroke after an HHS attack increased by 80% after adjusting for other cerebrovascular risks.

Several possible pathophysiological processes may explain the unexpectedly high risk of ischemic stroke observed after treatment for HHS. First, we hypothesize that a worse metabolic profile among the HHS group (compared to the non-HHS group) may partially explain the increased incidence of stroke observed during the one year follow-up in our study. In a study of HHS patients in Taiwan, poor compliance with medication (21.0%) and undiagnosed DM (10.9%) were the second and third most common precipitating factors in HHS [19]. A second theory links attacks of HHS in DM patients with marked fluctuations in glucose levels. Intermittent hyperglycemia may induce endothelial cell dysfunction and is associated with high oxidative stress [20]. Other studies have shown that fluctuations in glucose levels can enhance cell proliferation, induce the release of cytokines, and impair endothelial function, all of which contribute to the mechanisms underlying cerebrovascular events [21], [22]. Finally, among the HHS precipitating factors (such as infections, alcohol abuse, gastrointestinal bleeding, acute pancreatitis, silent myocardial infarction, mesenteric ischemia, use of certain medications, and noncompliance with diabetic treatment [23]), infection, in particular, is associated with an increased risk of ischemic stroke. Such infections include respiratory tract infections [24], tuberculosis [25], herpes zoster attacks [26], chronic Helicobacter pylori infections [27], and chronic hepatitis C infections [28]. Despite adjusting for several well-known cerebrovascular risk factors, the general health condition of our patients could not be inferred from the database. Therefore, the possibility that development of ischemic stroke was associated with an underlying infection or other stressful condition cannot be excluded.

Our study had several additional limitations related to the NHIRD database. The diagnoses of HHS, DM, ischemic stroke, and any other comorbid conditions were dependent on ICD codes from the NHIRD database. The National Health Insurance Bureau of Taiwan, however, has made every effort to verify the accuracy of diagnosis based upon random chart review and patient interview [29]. In fact, since inclusion criteria involved hospitalized type 2 DM patients, the dataset used in our study was the most accurate available. An additional limitation of the NHIRD database was its lack of information on tobacco use, dietary habits, physical activity, metabolic profiles, and body mass index, or the status of DM control, which also may be risk factors for ischemic stroke among DM patients. Nonetheless, given the magnitude and statistical significance of the observed effects in this study, these limitations were unlikely to have compromised our results. In fact, the strength of our study lies in its large sample size. In addition, because the National Health Insurance Program has 99% coverage in Taiwan, our study had minimal risk of selection bias.

In conclusion, this is the first study to investigate the risk of initial ischemic stroke in type 2 DM patients after HHS. It revealed that type 2 DM patients with HHS had an increased risk for developing ischemic stroke. It was not surprising that cardiovascular comorbidities, such as hypertension, coronary artery disease, atrial fibrillation, and chronic renal failure, were found to be important predictors for developing future ischemic stroke after HHS treatment. Therefore, interventions aimed at stroke prevention are extremely important in patients recovering from HHS who have such comorbidities.
